# Evaluate the performance of four artificial intelligence‐aided diagnostic systems in identifying and measuring four types of pulmonary nodules

**DOI:** 10.1002/acm2.13142

**Published:** 2020-12-24

**Authors:** Ming‐yue Wu, Yong Li, Bin‐jie Fu, Guo‐shu Wang, Zhi‐gang Chu, Dan Deng

**Affiliations:** ^1^ School of Public Health and Management Chongqing Medical University Chongqing China; ^2^ Department of Radiology The First Affiliated Hospital of Chongqing Medical University Chongqing China

**Keywords:** artificial intelligence, lung phantom, pulmonary nodules

## Abstract

**Purpose:**

This study aims to evaluate the performance of four artificial intelligence‐aided diagnostic systems in identifying and measuring four types of pulmonary nodules.

**Methods:**

Four types of nodules were implanted in a commercial lung phantom. The phantom was scanned with multislice spiral computed tomography, after which four systems (A, B, C, D) were used to identify the nodules and measure their volumes.

**Results:**

The relative volume error (RVE) of system A was the lowest for all nodules, except for small ground glass nodules (SGGNs). System C had the smallest RVE for SGGNs, −0.13 (−0.56, 0.00). In the Bland–Altman test, only systems A and C passed the consistency test, *P* = 0.40. In terms of precision, the miss rate (MR) of system C was 0.00% for small solid nodules (SSNs), ground glass nodules (GGNs), and solid nodules (SNs) but 4.17% for SGGNs. The comparable system D MRs for SGGNs, SSNs, and GGNs were 71.30%, 25.93%, and 47.22%, respectively, the highest among all the systems. Receiver operating characteristic curve analysis indicated that system A had the best performance in recognizing SSNs and GGNs, with areas under the curve of 0.91 and 0.68. System C had the best performance for SGGNs (AUC = 0.91).

**Conclusion:**

Among four types nodules, SGGNs are the most difficult to recognize, indicating the need to improve higher accuracy and precision of artificial systems. System A most accurately measured nodule volume. System C was most precise in recognizing all four types of nodules, especially SGGN.

## 1 Introduction

Artificial intelligence (AI) is increasingly used in image processing. Improvements in and combinations of various optimization methods are gradually being applied to various medical image processing fields. These include assisted localization of pulmonary nodules^1^ and digital tomography.^2^ The emergence of an artificial intelligence‐aided diagnostic system (AIADS) has been a great step forward in precision medicine. For instance, multislice spiral computed tomography (MSCT) can be combined with AIADS to measure the size, volume, and density of pulmonary nodules, aiding in systematic and rational clinical decision‐making and treatment.^3^, ^4^

However, the accuracy and precision of nodule volume measurement by AIADS is affected by several factors, including acquisition and reconstruction parameters, pulmonary nodule characteristics, and system technology.^5^, ^6^ This is a relatively young area of research requiring quantification of the impact of these factors on pulmonary nodule volume measurement. Limited research has focused on the influence of acquisition and reconstruction parameters on volume measurement.^7^, ^8^, ^9^, ^10^ A few studies have compared the accuracy of two detection systems for pulmonary nodule volume measurement.^11^ However, there are few reports comparing different AIADS to assess the influence of pulmonary nodule characteristics on the accuracy and precision of nodule measurement and detection.

The malignant potential of a pulmonary nodule varies depending on its density and size. Nodule diameter is strongly correlated with malignancy. Less than 1% of nodules with a diameter <5 mm are malignant compared with 6% to 28% of those measuring 5–10 mm and 64%–82% of nodules >20 mm in diameter.^12^, ^13^, ^14^, ^15^, ^16^ A ground glass nodule (GGN) is reportedly more likely to be malignant than a solid nodule (SN).^17^ Before the development of MSCT, it was difficult to qualitatively assess small nodules and GGNs because of their small size, low density, and lack of specificity on imaging.^18^, ^19^ However, MSCT has substantially increased the detection rate of SNs and GGNs by manual identification.^20^ However, due to the numerous scanned slices generated by MSCT, even if only one organ is examined, clinicians face a huge workload in thoroughly examining each study. AI software is based on automatic extraction by the computer of data on pulmonary nodules that indicates their morphologic features. It intelligently detects the shape, edge, density, and size of nodules to improve the diagnostic efficiency and accuracy of medical images. Therefore, the application of AI software in medical imaging can not only reduce pressure on physicians but also, more importantly, aid in faster diagnosis and treatment for patients. Many studies have shown AI systems have the advantage over traditional diagnostic methods of efficiency in identifying and diagnosing pulmonary nodules.^3^, ^4^ This study analyzed the performance of different AIADS software to determine factors influencing the accuracy of the identification of various pulmonary nodules.

In our study, the models of solitary pulmonary nodules were implanted into a commercial lung phantom. Scanned and reconstructed MSCT images were then analyzed by four different AIADSs and their performance compared. The purpose was to provide some information that might aid in technical improvement and in the clinical application of AIADSs.

## 2 MATERIALS AND METHODS

### 2.1 Lung phantom

A professional phantom (Multipurpose Chest Phantom N1 Lungman, Kyoto Kagaku, Japan) was used to simulate the chest of an adult male. Fifteen nodules with different diameters and densities were used to simulate pulmonary nodules in the chest. These included a small ground glass nodule (SGGN, diameter 5.33 ± 2.18 mm, density ‐800 HU or ‐630 HU), small solid nodule (SSN, diameter 5.33 ± 2.18 mm, density 100 HU), ground glass nodule (GGN, diameter 11 ± 1.1 mm, density ‐800 HU or ‐630 U), and solid nodule (SN, diameter 11 ± 1.1 mm, density 100 HU).

**Fig. 1:**
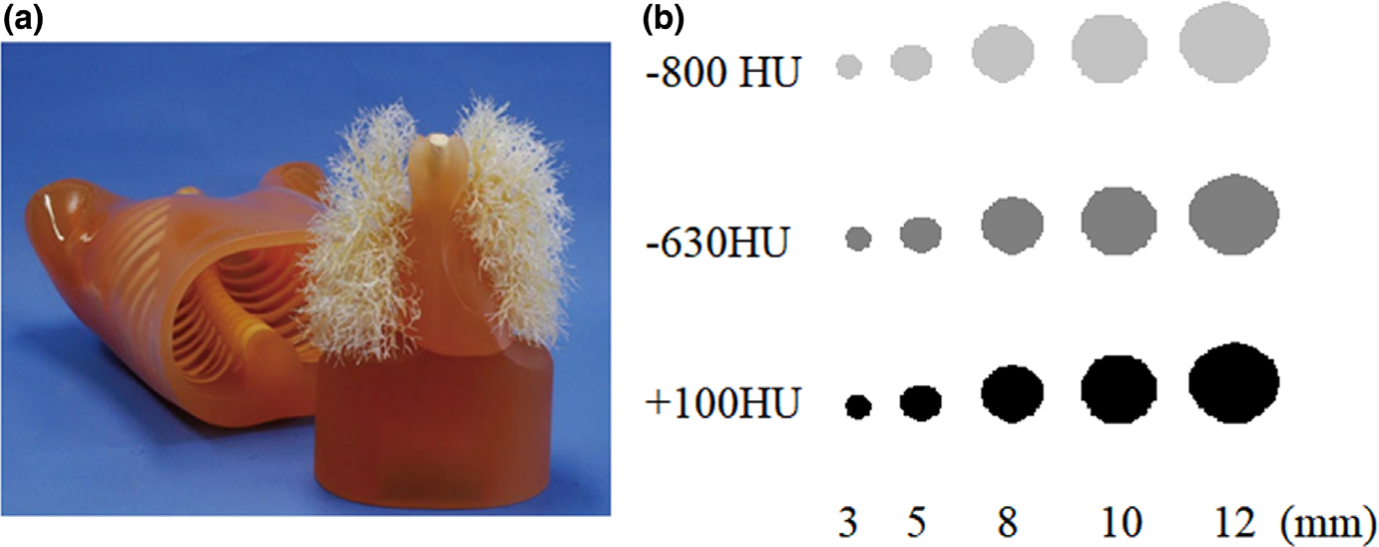
(a) The image of the professional phantom; (b) The diagram of nodules. Nodules are randomly distributed in the phantom. −800HU, −630HU, and + 100HU were the density of nodules. And 3, 5, 8, 10, 12mm were the diameter of nodules.

The phantom image and diagram of nodules are shown in Fig. 1.

**Fig. 2:**
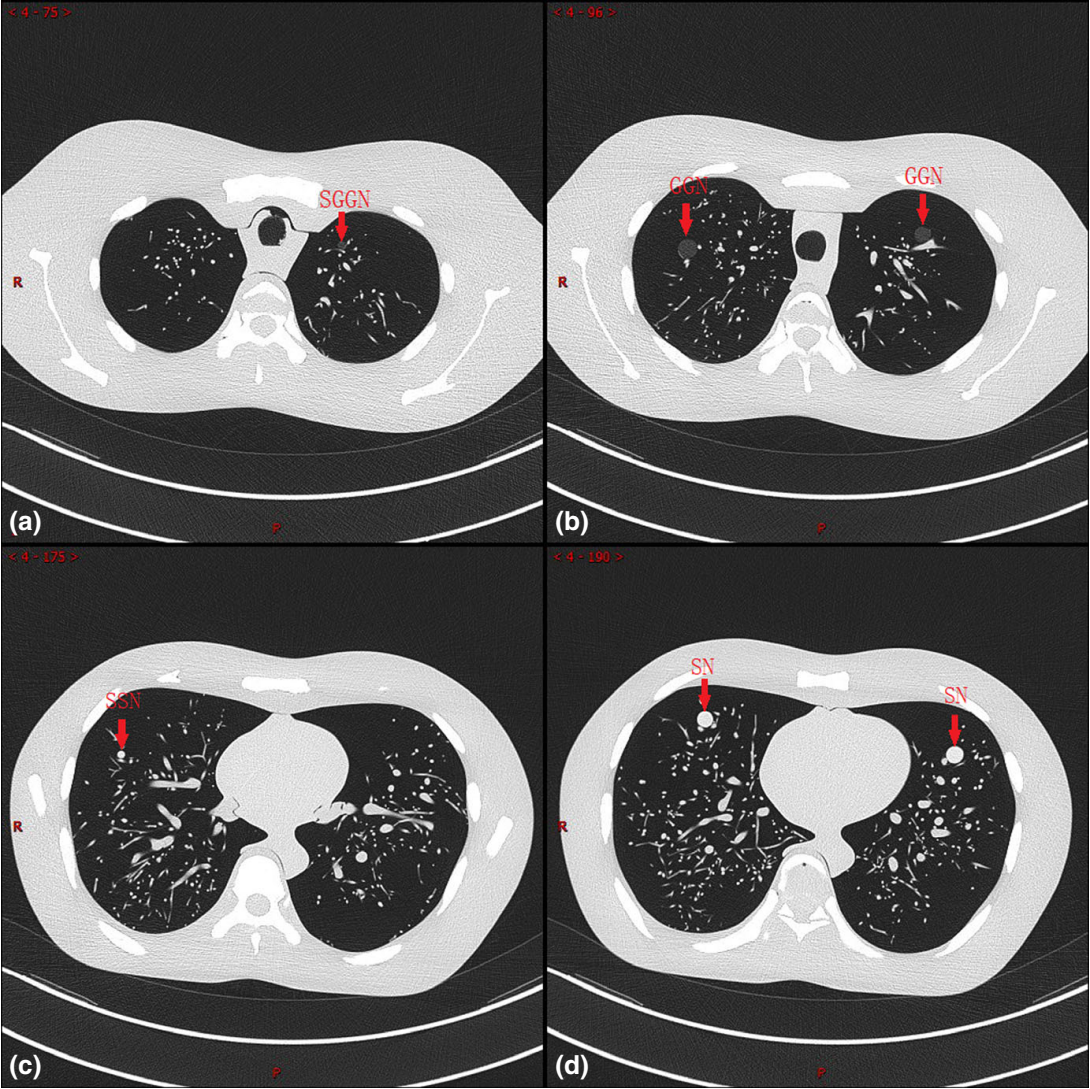
CT images of different types of nodules in phantom.

Partial CT images obtained by phantom scanning are shown in Fig. 2, showing four types of nodules in different scanning layers.

### 2.2 MSCT parameters

SIEMENS SOMATOM Definition Flash was used for scanning the phantom at 120 kVp with 100 mAs. The calculated effective dose was regular with 3.28 mSv. Other conventional acquisition parameters included scanning slice thickness 5 mm, rotation time 0.5 s, pitch 1.0, detector collimation 128 × 0.6 mm, field of view 360 mm, and pixel 512 × 512. A convolution kernel (B60f, sharp) was used to reconstruct the images in 1 mm slices. Considering the possible random error, the phantom was scanned repeatedly for three times. Finally, three groups of images were transmitted to PACS.

### 2.3 Four AI systems

Four AI pulmonary nodule‐assisted detection systems which were relatively mainstream and mature in China (Care.ai CT, YITU; σ‐Discover, 12Sigma; InferRead CT, Infervision; Lung‐Sight, IMsight) were used for image analysis and automatic detection of lung nodules. To maintain business privacy, we use A,B,C,D to name the four systems.

Care.ai CT: Based on excellent algorithm models and technologies such as 3D RetinaNet, the chest CT artificial intelligence aided diagnosis system is developed to realize all bit multitask intelligent diagnosis.

σ‐Discover: Through the combination of medical big data and deep learning technology, the bottleneck of medical field completely relying on doctor's experience and manual processing has been overturned and solved.

InferRead CT: Based on deep learning technology, it can trace back the historical images and intelligently match the historical cases similar to the current cases in the case database. So as to provide intelligent reference for doctors to make accurate clinical decisions.

Lung‐Sight: After deep learning of hundreds of thousands of clinical data and quantitative analysis of dozens of parameters of nodules, more than 3mm of nodules can be detected.

### 2.4 Image analysis

Two senior radiologists specializing in chest imaging used four AIADSs from different companies for image recognition and automatic detection of pulmonary nodules. The diameter of the implanted nodules was used to determine the true volume. Two radiologists recorded the volume detection data for each pulmonary nodule in each group of images by four different systems. After detection, they compared the consistency of their records with each other. If there were discrepancies in the data, then the nodules were redetected until the results were consistent.

### 2.5 Outcome measures

The results for each system’s performance for each type of nodule were compared in terms of the relative volume error (RVE) and miss rate (MR). These were calculated with the following formulas:

$$〈RVE=\frac{V_{M}‐V_{T}}{V_{T}}〉.$$

$$〈MR=\frac{N_{L}}{N_{T}}\times 100\%〉.$$

Relative volume error was defined as the ratio of the difference between the measured value and the reference value to the reference value. MR was defined as the ratio of undetected nodules to total nodules. Accuracy was defined as the best nodule volume measurement and was evaluated by the RVE, consistency test followed. Precision, defined as correctly identifying a nodule, was evaluated with the miss rate (MR), and receiver operating characteristic (ROC) curves.

### 2.6 Statistical analysis

The Kruskal–Wallis test was used for intergroup comparisons and the paired comparisons Kruskal–Wallis test for intragroup comparisons. The Bland–Altman test was used to assess the consistency of four AIADS. To compare the MR among the groups, the Chi‐square test or Fisher's exact probability test was used. Conventional ROC curves are used to represent dichotomous classifier performance. For multiclassification ROC curves, "One vs all" is used.^21^ The diagnostic performance for the four types of nodules was analyzed by one vs all ROC curves, calculating the area under the curve (AUC), sensitivity, and specificity of each system. The Z‐test was used to paired comparison of AUC. SPSS 20.0 was used to analyze differences in RVE and MR. The Bland–Altman test and one vs all ROC curve analysis were carried out in Medcalc. GraphPad Prism 5.01 was used for statistical analysis. The significance level was set at 0.05.

## 3 Results

### 3.1 Accuracy of the four systems for measuring nodule volume

**Fig. 3:**
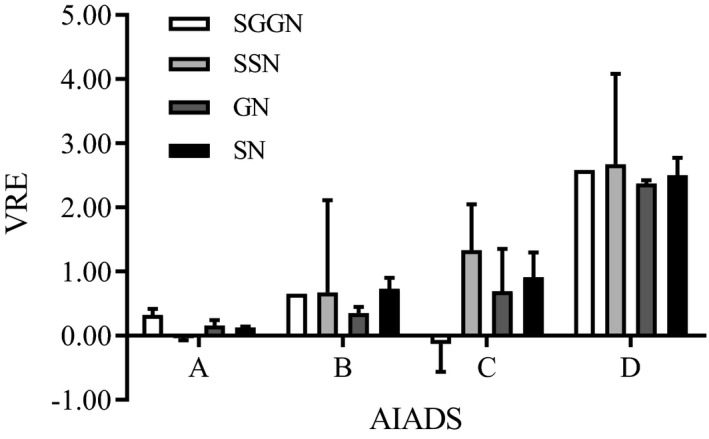
RVE of each AIADS for detecting different nodules. RVE, the relative volume error; AIADS, artificial intelligence‐aided diagnosis system; SGGN, small ground glass nodule; SSN, small solid nodule; GGN, ground glass nodule; SN, solid nodule; A,B,C,D is the code name of four AIADSs.

The RVE of system A was the lowest, while that of system D was the highest (Fig. 3). For SGGNs there were significant differences when comparing the systems with each other as follows: C < A < B < D were − 0.13 (−0.56, 0), 0.32 (0.14, 0.42), 0.65 (0.65, 0.65), 2.58 (2.58, 2.58), respectively (Table 1). For SSNs, the RVE of system A was − 0.04 (−0.07,−0.04), significantly lower than that of the other systems. There were no significant differences between of B, C, D. For GGNs, there were significant differences when comparing two systems: A < C < B < D with results of 0.16 (0.03, 0.24), 0.69 (0.27, 1.35), 0.35 (0.28, 0.45), and 2.37 (2.37, 2.42), respectively. For SNs, there were significant differences between two systems except between systems B and C. The ranking was A < B < C < D, equal to 0.13 (0.10, 0.14), 0.73 (0.43, 0.90), 0.91 (0.48, 1.30), and 2.50 (2.22, 2.77), respectively.

**Table 1:** Paired Comparisons of Nodules’ RVE for Four AIADSs

Nodule groups	A vs B		A vs C		A vs D		B vs C		B vs D		C vs D	
	Statistics	Sig.	Statistics	Sig.	Statistics	Sig.	Statistics	Sig.	Statistics	Sig.	Statistics	Sig.
SGGN	−5.37	<0.01*	8.08	<0.01*	−16.63	<0.01*	13.45	<0.01*	−11.26	<0.01*	−24.71	<0.01*
SSN	−8.28	<0.01*	−8.00	<0.01*	−9.95	<0.01*	0.28	>0.99	−1.68	0.56	−1.95	0.30
GGN	−5.69	<0.01*	−8.60	<0.01*	−19.46	<0.01*	−2.91	0.02*	−13.77	<0.01*	−10.86	<0.01*
SN	−6.69	<0.01*	−8.05	<0.01*	−15.29	<0.01*	−1.36	>0.99	−8.60	<0.01*	−7.24	<0.01*

Note

The Kruskal–Wallis test was used.

Abbreviations: GGN, Ground Glass Nodules; RVE, relative volume error; SGGN, Small Ground Glass Nodules; SN, Solid Nodules; SSN, Small Solid Nodules.

* Significant < 0.05.

**Fig. 4:**
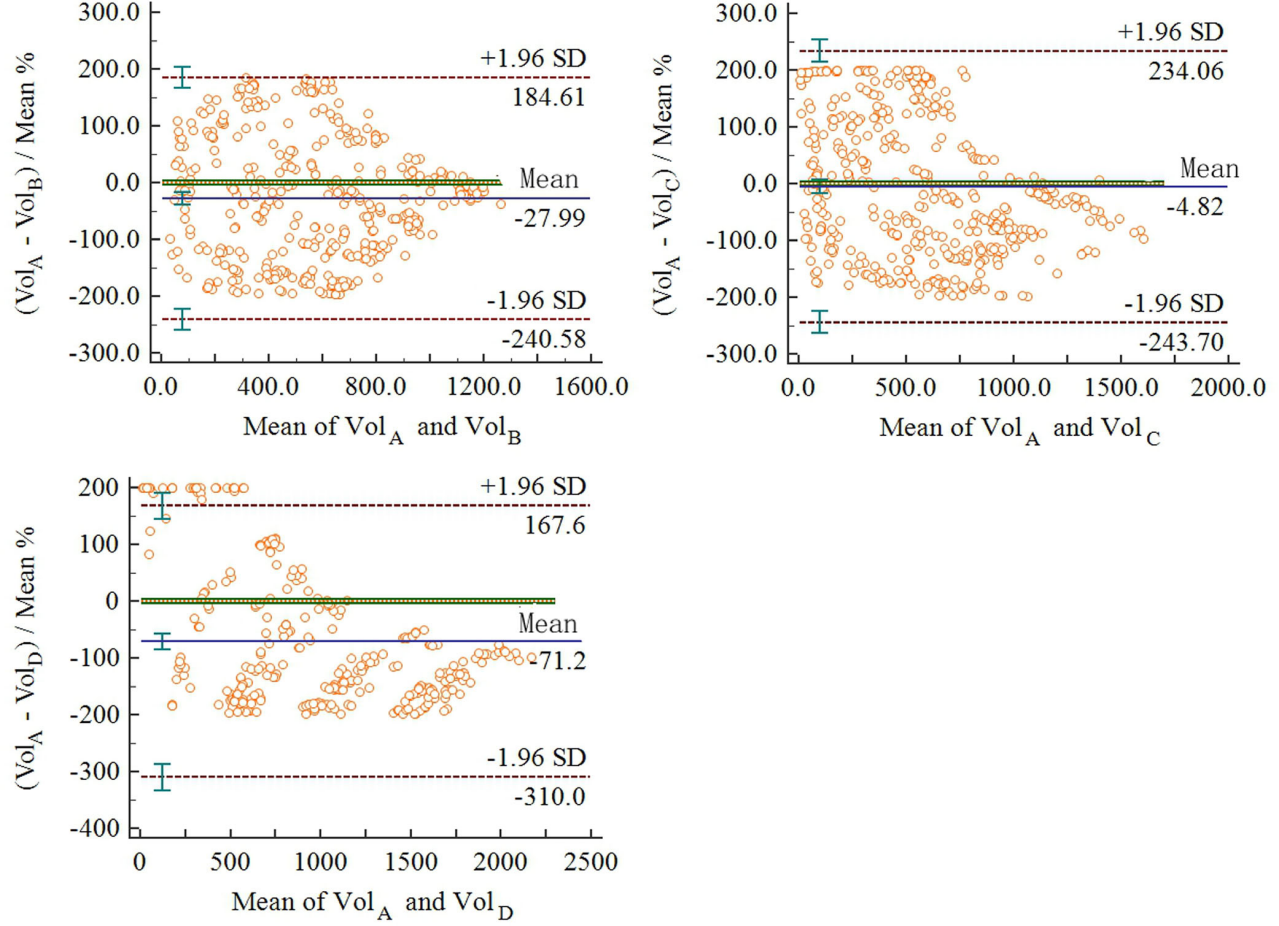
Consistency Test between system A and others. A,B,C,D is the code of four AIADSs.

In terms of RVE, system A has the highest accuracy; hence, we used it as the standard with which to compare the other systems (Fig. 4). Systems A and C passed the consistency test, *P* = 0.40, indicating that A and C have similar accuracy in measuring nodule volume. However, compared with A, systems B and D did not pass the consistency test, *P* < 0.01.

### 3.2 Precision of the four systems in identifying different types of nodule

All four systems correctly identified the SN, but the results for the SGGN and SSN were fairly different. System C had the lowest MR for SGGN and SSNs, equal to 4.17% and 0.00%, while other systems differed significantly. It was performing significantly better than the other systems. For the GGN, system D had a significantly higher MR than the other systems. On the other hand, all four systems correctly identified the SN at a rate of 100% (Table 2).

**Table 2:** The Nodules’ MR of AIADSs and the Result of Paired Comparisons

Nodule groups	MR(%)				A vs B		A vs C		A vs D		B vs C		B vs D		C vs D	
	A	B	C	D	Statistics	Sig.	Statistics	Sig.	Statistics	Sig.	Statistics	Sig.	Statistics	Sig.	Statistics	Sig.
SGGN	25.93	60.19	4.17	71.30	51.70	<0.01*	40.00	<0.01*	89.00	<0.01*	155.30	<0.01*	5.92	0.02*	207.15	<0.01*
SSN	33.33	5.56	0.00	25.93	26.61	<0.01*	43.20	<0.01*	1.42	0.23	Fisher	0.03*	16.90	<0.01*	32.17	<0.01*
GGN	0.69	4.17	0.00	47.22	Fisher	0.12	Fisher	0.99	85.56	<0.01*	Fisher	0.03*	69.91	<0.01*	89.02	<0.01*
SN	0.00	0.00	0.00	0.00	——	——	——	——	——	——	——	——	——	——	——	——

Notes

The Chi‐square test or Fisher's exact probability test was used.

Abbreviations: GGN, Ground Glass Nodules; MR, Missed Rate;SGGN, Small Ground Glass Nodules; SN, Solid Nodules, A,B,C,D is the code of four AIADSs; SSN, Small Solid Nodules .

* Significant < 0.05.

**Fig. 5:**
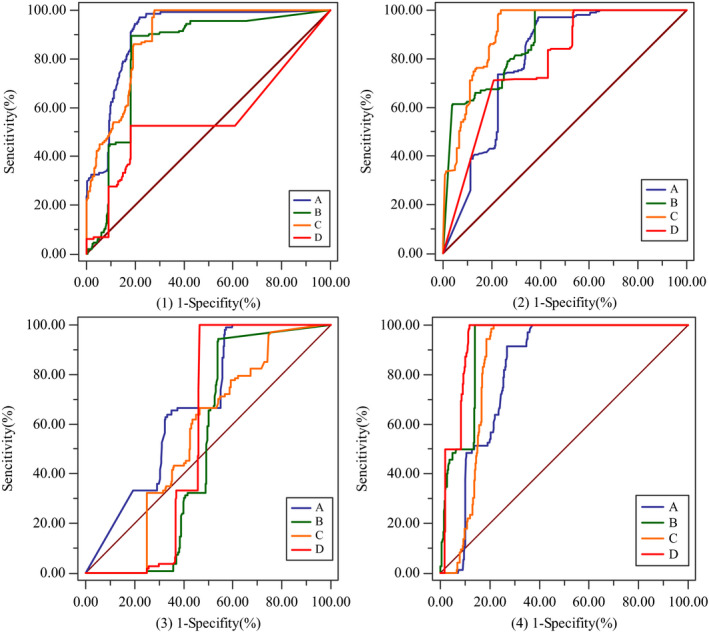
The one vs all ROC curves. (1) Dichotomies of SGGN and others; (2) Dichotomies of SSN and others; (3) Dichotomies of GGN and others; (4) Dichotomies of SN and others; A,B,C,D is the code of four AIADSs.

According to ROC curve analysis, one vs all relationships existed between of four nodules, which were SGGN and others, SSN and others, GGN and others, SN and others. Overall, the diagnostic performance of systems A, B, and C for nodules (≧10 mm) was generally better, and all AUCs were >0.80. System D performed worse in classifying the SGGN (AUC 0.79), SSN (0.57), and GGN (0.56) (Fig. 5).

For the SGGN, system C’s performance was significantly superior to the other three. Its AUC was 0.91, with a sensitivity of 100.00% and a specificity of 76.20%. None of the systems did well with the SSN, with all AUCs close to 0.50. System A, however, did perform significantly better even for this lesion (AUC 0.68, sensitivity 99.10%, and specificity 61.60%). For the GGN, systems A and C performed significantly better than B and D. For the SN, system D performed better than the other three (AUC 0.94, sensitivity 100.00%, and specificity 88.00% (Tables 3 and 4).

**Table 3:** The related index of one versus all ROC Analysis

Dichotomies	A			B			C			D		
	AUC	Sen%	Spec%	AUC	Sen%	Spec%	AUC	Sen%	Spec%	AUC	Sen%	Spec%
(1)	0.80 (0.76, 0.83)	97.20	60.80	0.88 (0.85, 0.90)	100.00	62.30	0.91 (0.89, 0.94)	100.00	76.20	0.79 (0.75, 0.82)	71.30	79.00
(2)	0.68 (0.63, 0.72)	99.10	61.60	0.52 (0.46, 0.57)	99.10	53.90	0.55 (0.50, 0.60)	96.30	45.10	0.57 (0.52, 0.62)	100.00	51.40
(3)	0.91 (0.88, 0.93)	97.20	78.00	0.83 (0.79, 0.86)	89.60	81.60	0.89 (0.86, 0.92)	100.00	72.00	0.56 (0.52, 0.60)	52.80	81.80
(4)	0.82 (0.79, 0.85)	91.70	72.90	0.92 (0.89, 0.94)	100.00	85.90	0.86 (0.82, 0.88)	100.00	78.40	0.94 (0.92, 0.96)	100.00	88.00

Note:

One vs all ROC Analysis was used; (1) Dichotomies of SGGN and others; (2) Dichotomies of SSN and others; (3) Dichotomies of GGN and others; (4) Dichotomies of SN and others; A,B,C,D is the code of four AIADSs.

**Table 4:** Paired Comparison of AUC for One vs all ROC Analysis

Dichotomies	A vs B		A vs C		A vs D		B vs C		B vs D		C vs D	
	Statistics	Sig.	Statistics	Sig.	Statistics	Sig.	Statistics	Sig.	Statistics	Sig.	Statistics	Sig.
(1)	6.44	<0.01*	11.09	<0.01*	0.49	0.62	3.35	<0.01*	5.71	<0.01*	6.75	<0.01*
(2)	3.14	<0.01*	2.27	0.02*	2.02	0.04*	1.98	0.05*	5.50	<0.01*	0.95	0.34
(3)	6.07	<0.01*	1.16	0.24	11.97	<0.01*	4.07	<0.01*	9.03	<0.01*	9.52	<0.01*
(4)	9.02	<0.01*	2.06	0.04*	8.86	<0.01*	4.86	<0.01*	2.53	0.01*	5.54	<0.01*

Note:

The Z‐test was used.

(1) Dichotomies of SGGN and others; (2) Dichotomies of SSN and others; (3) Dichotomies of GGN and others; (4) Dichotomies of SN and others; A,B,C,D is the code of four AIADSs.

* Significant < 0.0.5.

In summary, compared with other systems, system A was best at classifying SSNs and GGNs. System C was best for the SGGN, and system D was best at classifying the SN but performed poorly with the other three types of nodules.

## 4 Discussion

With the development of MSCT technology, subsecond scanning speed and submillimeter scanning volume are increasingly used. It can detect a variety of microlesions, more clearly indicating their morphologic characteristics. The international manufacturers of imaging equipment have launched their own semiautomatic or automatic software for assessing pulmonary nodules.^12^, ^16^ In this study, we evaluated the accuracy and precision of the four latest AIADSs introduced in China, finding that the performance of the systems varied depending on the size and density of the nodules.

The factors that may affect the detection and evaluation of pulmonary nodule detection include scanning and reconstruction parameters, the character of the nodules, and technology used for measurements. Many studies have shown that radiation dose has no significant effect on the measurement of a nodule’s diameter, nor is measurement error affected by the reconstruction algorithm used.^22^, ^23^, ^24^, ^25^, ^26^, ^27^, ^28^ A few studies, however, have indicated a reconstruction algorithm that reduces measurement errors for GGNs but not for SNs.^11^

In terms of the accuracy of the AIADSs we assessed, RVE with A was lowest overall, indicating its greater accuracy. System C most precisely identified SGGNs. System D was least accurate in measuring volume. Therefore, systems A and C were most accurate for measuring pulmonary nodule volume. The core technology for this measurement by AI software is nodule segmentation. If the segmentation is accurate, then the volume measurement is accurate. Therefore, emphasis on nodule segmentation technology and the establishment of mathematical models for the nodules of different sizes and densities might help improve less accurate systems, such as B and D.

Even experienced chest radiologists find it challenging to classify pulmonary nodules, with poor consistency in observer results. If management guidelines for pulmonary nodules are based only on the size and classification, then inconsistencies in the classification of will lead to inconsistencies in the management.^29^, ^30^ To address this issue, the diagnosis of nodule type must be more objective. CT manufacturers and software developers must improve the algorithms and technologies to achieve this goal.

In our study, we analyzed the abilities of the AIADSs to recognize four types of nodules. The MR of system C was significantly lower than of other systems. System C was more sensitive in recognizing small nodules and low‐density nodules. All four systems did well at recognizing SNs, likely because they are larger and have higher density. By contrast, SGGNs are more difficult to identify. Due to its low density, small diameter and unclear boundary, the SGGNs may not be as clear as SNs compared with the lung background, so the software has certain difficulties in recognition. These results are consistent with that of Reeves et al.^31^

We found significant differences between the AIADSs we analyzed. Although one or another system had the best performance for a particular purpose such accuracy in measuring nodule volume (e.g., system A) or precision in identifying the type of nodule (such as system C) for SGGNs, no one system consistently outperformed the others in all the aspects of pulmonary nodule assessment. The shortcomings we identified, particularly for systems B and D, might prompt the developers to improve the algorithms to achieve better performance. Many studies have focused on various methodologies to distinguish among the types of pulmonary nodules, such as the support vector machine,^32^ neural networks,^33^ decision trees,^34^ or other classifiers,^35^ but the results have been unsatisfactory. A study based on a deep residual neural network yielded a good results, indicating that combining deep residual learning, course learning, and transfer learning can improve the accuracy of nodule classification.^36^

## 5 Conclusion

Among four types nodules, SGGNs are the most difficult to recognize, indicating the need to improve higher accuracy and precision of artificial systems. System A most accurately measured nodule volume. System C was most precise in recognizing all four types of nodules, especially SGGN. The superior performance of the software is related to its stronger computing power and more mature algorithms. This paper is helpful to provide reference for quantitative selection of better software for clinical selection.

## Conflict of Interest

The authors declare they have no competing interests.

## Author contribution

Ming‐yue Wu and Yong Li analyzed the data and wrote the manuscript; Bin‐jie Fu and Guo‐shu Wang collected data and participated in manuscript revision; Zhi‐gang Chu gave the fund assistance; As corresponding author, Dan Deng was mainly responsible for the revision of the manuscript. All authors have read and approved the final manuscript.
